# Heterogeneity in Response to MCT and Psychoeducation: A Feasibility Study Using Latent Class Mixed Models in First-Episode Psychosis

**DOI:** 10.3390/healthcare10112155

**Published:** 2022-10-28

**Authors:** Marta Ferrer-Quintero, Daniel Fernández, Raquel López-Carrilero, Luciana Díaz-Cutraro, Marina Verdaguer-Rodríguez, Helena García-Mieres, Elena Huerta-Ramos, Juana Gómez-Benito, Trini Peláez, Irene Birulés, Ana Barajas, Esther Pousa, Marisa Barrigón, Alfonso Gutiérrez-Zotes, Eva Grasa, Isabel Ruiz-Delgado, Esther Lorente-Rovira, Jordi Cid, on behalf of the Spanish Metacognition Study Group, Susana Ochoa

**Affiliations:** 1 Parc Sanitari Sant Joan de Déu, Sant Boi de Llobregat, 08830 Barcelona, Spain; 2 Department of Social and Quantitative Psychology, Faculty of Psychology, University of Barcelona, 08035 Barcelona, Spain; 3 Centro de Investigación Biomédica en Red de Salud Mental, Instituto de Salud Carlos III, 28029 Madrid, Spain; 4 Serra Húnter Fellow, Department of Statistics and Operations Research (DEIO), Universitat Politècnica de Catalunya, 08028 Barcelona, Spain; 5 Institute of Mathematics of UPC—BarcelonaTech (IMTech), 08028 Barcelona, Spain; 6 Sant Joan de Déu Research Foundation, 08950 Esplugues de Llobregat, Spain; 7 COMSAL Research Group, Psychology Department, FPCEE Blanquerna, Ramon Llull University, 08022 Barcelona, Spain; 8 Departament de Psicologia Clínica i de la Salut, Facultat de Psicologia, Universitat Autònoma de Barcelona, 08193 Bellaterra, Spain; 9 Health Services Research Group, Instituto Hospital del Mar de Investigaciones Médicas, 08003 Barcelona, Spain; 10 Centro de Investigación Biomédica en Red de Epidemiología y Salud Pública, Instituto de Salud Carlos III, 28029 Madrid, Spain; 11 Departamento de Medicina y Ciencias de la Vida, Universitat Pompeu Fabra, 08003 Barcelona, Spain; 12 Grupo de Estudios de Invariancia de la Medida y Análisis del Cambio (GEIMAC), Institut de Neurociències, Universitat de Barcelona, 08035 Barcelona, Spain; 13 Serra Húnter Fellow Departament de Psicologia Clínica i de la Salut, Facultat de Psicologia, Universitat Autònoma de Barcelona, 08193 Bellaterra, Spain; 14 Department of Research, Centre d’Higiene Mental Les Corts, 08029 Barcelona, Spain; 15 Department of Psychiatry, Hospital de la Santa Creu i Sant Pau, Institut d’Investigació Biomèdica-Sant Pau (IIB-Sant Pau), 08025, Barcelona, Spain; 16 Departament of Psychiatry, University Hospital Virgen del Rocio, 41013 Seville, Spain; 17 Hospital Universitari Institut Pere Mata, Institut d’Investigació Sanitària Pere Virgili, CERCA, Universitat Rovira i Virgili, Reus, Spain, 43206 Reus, Spain; 18 Unidad de Salud Mental Comunitaria Malaga Norte, 29014 Málaga, Spain; 19 Psychiatry Service, Hospital Clínico Universitario de Valencia, 46010 Valencia, Spain; 20 Mental Health & Addiction Research Group, IdiBGi, Institut d’Assistencia Sanitària, 17190 Girona, Spain

**Keywords:** first-episode psychosis, social cognition, metacognition, latent class mixed models, metacognitive training, psychoeducation

## Abstract

Metacognitive training (MCT) is an effective treatment for psychosis. Longitudinal trajectories of treatment response are unknown but could point to strategies to maximize treatment efficacy during the first episodes. This work aims to explore the possible benefit of using latent class mixed models (LCMMs) to understand how treatment response differs between metacognitive training and psychoeducation. We conducted LCMMs in 28 patients that received MCT and 34 patients that received psychoeducation. We found that MCT is effective in improving cognitive insight in all patients but that these effects wane at follow-up. In contrast, psychoeducation does not improve cognitive insight, and may increase self-certainty in a group of patients. These results suggest that LCMMs are valuable tools that can aid in treatment prescription and in predicting response to specific treatments.

## 1. Introduction

People that experience a first episode of psychosis (FEP) have highly variable outcomes, which range from sustained remission to treatment resistance from onset [1]. Despite efforts in identifying early treatment strategies [2], it is still a clinical challenge to deliver optimized treatment to prevent relapse and functional decline [3].

Cognitive behavioral models of psychosis have fostered the development of psychological interventions that target the cognitive biases involved in the genesis and maintenance of psychosis [4]. These have been studied as promising treatments for psychosis because in the past decades, despite considerable advances in pharmacological treatment, the outcomes of psychosis have not improved significantly [5].

Metacognitive training (MCT) [6] has emerged as one of the most effective psychological treatments for positive symptoms of psychosis [7]. MCT combines psychoeducation, cognitive bias modification, and strategy teaching to correct data-gathering biases. This approach aims to sow the seeds of doubt [8,9] by considering cognitive biases as a deviation from normality. Systematic-review findings have supported its efficacy in improving positive and negative symptoms, self-esteem, and functioning in schizophrenia [7,10], and recent studies have proven that it is also a valid intervention for people with FEP [11].

These broad beneficial effects may be rooted in that MCT intervenes over most of the cognitive biases and cognitive constructs that have established evidence of their importance in the genesis of psychosis, its maintenance, and outcomes. MCT includes sessions that work on domains of social cognition such as facial emotion recognition and theory of mind, both of which are strong predictors of outcome [12]. Likewise, it targets the jumping-to-conclusions bias (JTC), which has been repeatedly associated with delusions, poorer neurocognition, and measures of outcome [13,14,15,16,17].

A consistent finding in the literature is that MCT improves cognitive insight by increasing self-reflectivity and reducing self-certainty [18], the two domains that compose cognitive insight. Cognitive insight refers to the set of cognitive processes that permit questioning one’s beliefs and appraisals and reevaluating anomalous experiences or misinterpretations [19]. Self-reflectivity refers to a person’s ability for introspection and willingness to admit fallibility. Conversely, self-certainty refers to the confidence a person has in their beliefs and judgments [19]. It is suggested that the formula for good cognitive insight is high self-reflectivity and low self-certainty [20]. This is because self-reflectivity has usually been associated with better outcomes and treatment response [21], while self-certainty is associated with more delusions and worse cognitive function [22,23].

Although meta-analytic findings have supported the efficacy of MCT in psychosis [7,24], this is usually at a medium effect size [24]. However, this seems to be the case for most effective psychological interventions for psychosis [25]. One reason for it may be that most clinical trials report averaged results, which blurs the vast heterogeneity in psychosis [26] and does not permit detecting the patients that indeed benefit from an intervention and their clinical characteristics. Furthermore, some of the MCT effects are apparent after a sleeper effect [27], but other patients need extended treatment to consolidate the effects [28]. Understanding the heterogeneity in response to MCT could point to better treatment strategies addressed to the specific characteristics of each patient. In particular, this is a vexing issue in first-episode psychosis because early targeted treatment may help promote recovery and prevent relapse [29].

Unsupervised learning methods such as latent class mixed models (LCMMs, also known as growth mixture models) are a useful approach for studying longitudinal trajectories of latent variables. LCMMs have the advantage of being able to capture inter-individual differences in intra-individual change over time while preserving the heterogeneity of the population [30]. Another characteristic of the LCMM method is that it is a data-driven method, which allows studying longitudinal changes of a variable as it occurs naturally.

Previous studies have used LCMMs to understand trajectories of illness or outcome [31,32], but [31,32] this method has not been used to explore trajectories of cognitive biases in response to different psychological interventions.

We sought to study the feasibility of using LCMMs to understand patterns of change in cognitive insight in individuals with FEP who have received MCT. To test whether this is a specific effect of MCT, we compared latent trajectories between patients that received MCT and a group of patients who received psychoeducation. Psychoeducation is a psychological intervention that has proven to be effective in promoting better social and functional outcomes [33], but according to the literature, psychoeducation does not seem to improve cognitive insight [34,35]. Thus, the psychoeducation group will help compare the effects of MCT as opposed to how cognitive insight changes naturally.

## 2. Materials and Methods

This study aims to provide proof-of-concept evidence of using LCMMs to understand patterns of change in response to psychotherapy. We conducted a secondary analysis of data from a blind, multicentric clinical trial that has been published elsewhere [11]. Briefly, the original study recruited 126 patients with FEP from nine participating mental health centers: Servicio Andaluz de Salud of Jaén, Málaga and Motril (Granada), Salut Mental Parc Taulí (Sabadell), Hospital de Santa Creu i Sant Pau (Barcelona), Centro de Higiene Mental Les Corts (Barcelona), Institut d’Assistència Sanitària Girona, Hospital Clínico Universitario de Valencia, and Parc Sanitari Sant Joan de Déu (coordinating center). Inclusion criteria were a diagnosis of schizophrenia, psychotic disorder not otherwise specified, delusional disorder, schizoaffective disorder, brief psychotic disorder, or schizophreniform disorder (according to DSM-IV-TR); (2) <5 years from the onset of symptoms; (3) a score of ≥3 in item delusions, grandiosity, or suspicions of PANSS in the previous year. Exclusion criteria were: traumatic brain injury, dementia, or intellectual disability (premorbid IQ ≤ 70); (2) substance dependence; and (3) PANSS ≥5 in hostile and uncooperativeness, or ≥6 in suspiciousness. This was chosen to avoid altering the dynamics of the group interventions. Participants were randomized to receive either MCT or psychoeducation. Psychoeducation included modules on healthy habits, risk behaviors, prevention of relapse, video forums, resources for work, leisure activities, and community resources. The original study randomized participants using blocks of four from a list of random numbers, and 55 patients were allocated to psychoeducation while 67 received MCT. The remaining participants were either excluded or declined to participate in the study. All participants were assessed at three points: baseline, post-treatment, and six-month follow-up.

The assessment included the following:

*Sociodemographic questionnaire:* Data on sociodemographic variables were collected on-site. Diagnosis and treatment were collected from the clinical history of the participants. We transformed the antipsychotic treatment to olanzapine defined daily dose (DDD) [36].

*Clinical measures:* The Positive and Negative Syndrome Scale (PANSS) [37,38] was used to measure symptom severity. The Spanish version of the Scale Unawareness of Mental Disorders (SUMD) [39,40] was used to measure unawareness of the mental disorder. Higher scores represent more unawareness of the mental disorder. We used the Rosenberg Self-Esteem Scale [41], where higher scores indicate better self-esteem.

*Metacognition:* The Beck Cognitive Insight Scale (BCIS) [20,42] was used to measure cognitive insight. The BCIS is composed of two subscales: self-certainty and self-reflectivity, which are analyzed separately. Higher scores in self-reflectivity represent more ability to question one’s beliefs. Higher scores in self-certainty represent more certainty in one’s interpretations and misinterpretations. The beads task [43] was used to measure the JTC. Participants were shown a picture of two containers filled with 100 colored beads in reciprocal proportions. We used three trials with different conditions: a probabilistic trial with an 85/15 ratio, a second probabilistic trial with a 60/40 ratio, and a final trial with an affective condition in a 60/40 ratio. Participants were told that the computer had selected a container and that the goal of the task was to determine which container. To this aim, participants were shown one bead at a time. The participant was instructed to see as many beads as they needed to guess what container the beads came from. Our outcome variable was the draws to decision in the three probabilistic conditions. Less than 3 draws to decision is considered indicative of presenting the JTC bias.

*Social cognition:* The Internal, Personal and Situational Attributions Questionnaire (IPSAQ) [44] was used to assess attributional style. We used two indexes: personalizing bias and externalizing bias. Personalizing bias refers to a tendency to blame others rather than circumstances for negative events. Externalizing bias refers to a tendency to attribute the causes of negative events to others or circumstances rather than to oneself [45]. The faces test [46,47] was used to measure emotion recognition. A reduced version of the hinting task [48,49] was used to measure theory of mind.

*Global functioning:* The Global Assessment of Functioning (GAF) [50] was used to measure global functioning on a scale of 0–100. Higher scores represent better functioning.

The Ethics Committee of each participating center approved this project.

LCMMs were conducted using the R package lcmm [51] from the statistical software R version 4.0.2 [52]. This unsupervised learning technique classifies individuals into groupings with similar trajectory patterns, called latent classes. Following the strategy of Nagin et al. [53], we fit the respective models for the outcomes of interest (self-reflectivity and self-certainty) at the three points of assessment, for which the time metric was the time at the assessment (baseline, post-treatment, and follow-up). The number of group trajectories was determined by analyzing 2–6 group models without covariates. Model selection to determine the optimal number of latent trajectories was performed according to the Bayesian information criterion (BIC), where a lower value indicates a better fit [54]. Average posterior probabilities above 70% were checked as well [55]. Individuals were allocated to classes according to maximum a posteriori criterium (MAP). To ensure adequate results, in this work we only included patients with complete data on self-reflectivity and self-certainty at the three points of assessment.

At baseline, the MCT and the psychoeducation groups were compared with chi-square tests for categorical variables and Student t-tests for continuous variables.

To compare trajectories, we used Mann–Whitney U tests, for which the effect size was reported with ranked biserial correlations.

## 3. Results

The final sample included 62 participants with complete data on self-reflectivity and self-certainty at the three points of assessment.

At baseline, the two groups only differed in that the MCT group had higher baseline global functioning than the psychoeducation group (t(60) = −2.857, *p* = 0.006). Table 1 presents baseline data of the two groups.

### 3.1. Cognitive Insight Trajectories in the MCT Group

**Figure 1:**
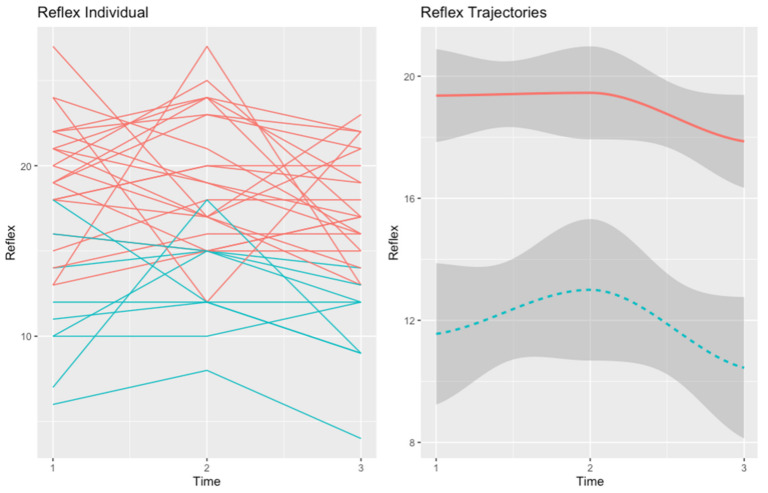
Trajectories of self-reflectivity in the MCT group. The red trajectory depicts the “High SR” trajectory (n = 20). The second trajectory (in blue), refers to “Improving SR” (n = 8).

**Figure 2:**
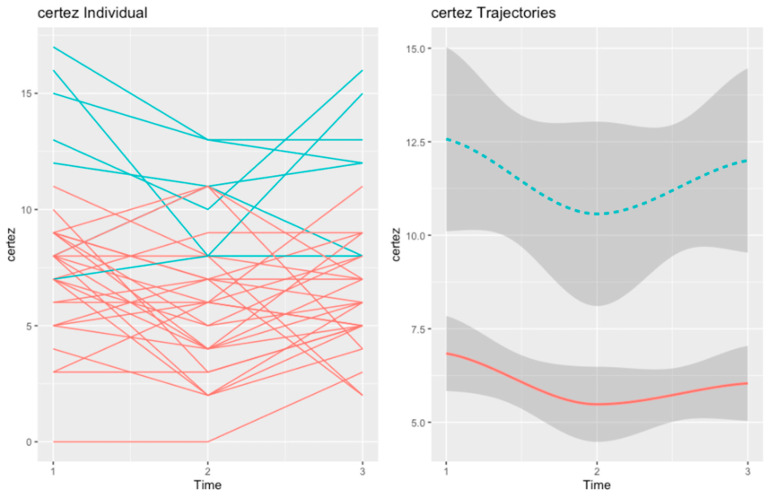
Trajectories of self-certainty in the MCT group: “Initial Decline” (in red, n = 22) and “Responsive SC” (in blue, n = 6).

We found two trajectories for self-reflectivity and two trajectories for self-certainty. The four trajectories are summarized in Figure 1 and Figure 2. Differences between each pair of trajectories at baseline, post-test, and follow-up can be found in Supplementary Table S1.

#### 3.1.1. Self-Reflectivity

We found a trajectory (in red), henceforth “High SR” (n = 20), that grouped patients with high baseline self-reflectivity. The “High SR” trajectory included patients whose self-reflectivity was stable during the baseline and post-treatment assessments but declined at follow-up. The second trajectory (in blue), named “Improving SR” (n = 8), grouped patients with low baseline self-reflectivity. The “Improving SR” trajectory presented an improvement in self-reflectivity at follow-up, which declined to a level below baseline at the 6-month follow-up. Figure 1 shows the graphical representations of the two trajectories of self-reflectivity in the MCT group.

At baseline, the two self-reflectivity trajectories differed in negative (U = 20.00, *p* = 0.002) and general (U = 22.00, *p* = 0.003) symptoms, global functioning (U = 23.00, *p* = 0.004), and self-esteem (U = 29.50, *p* = 0.011). Patients in the “High SR” trajectory presented better scores in all the variables, indicating a better clinical state at the moment of the assessment.

At post-test, the two trajectories still differed in negative symptoms (U = 12.00, *p* = 0.004), general symptoms (U = 21.50, *p* = 0.026), global functioning (U = 20.50, *p* = 0.021), and self-esteem (U = 27.00, *p* = 0.05). However, at this point of assessment, the “Improving SR” trajectory had significantly worse clinical insight (U = 24.00, *p* = 0.040).

Finally, at the 6-month follow-up, the two trajectories only differed in negative (U = 22.00, *p* = 0.03) and general symptoms (U = 10.00, *p* = 0.010) and clinical insight (U = 20.00, *p* = 0.020).

#### 3.1.2. Self-Certainty

For self-certainty, we also found two trajectories in the MCT group. The “Initial Decline” (in red, n = 22) trajectory grouped patients with moderate levels of self-certainty at baseline. At post-test, the scores in self-certainty declined, but they increased again to baseline levels at the 6-month follow-up.

The second trajectory (in blue, n = 6), henceforth “Responsive SC”, comprised patients with very high self-certainty at baseline. The scores in self-certainty presented a steep decline at post-test but increased again at follow-up. However, at follow-up, the scores in self-reflectivity did not reach baseline levels.

The two trajectories of self-certainty in the MCT group are depicted in Figure 2.

At baseline, the two trajectories of self-certainty did not differ in any variable. However, at post-test, the “Responsive SC” trajectory had worse positive symptoms (U = 18.50, *p* = 0.015). The difference in positive symptoms was maintained at follow-up (U = 20.50, *p* = 0.025), but patients in the “Initial Decline” trajectory also displayed worse general symptoms (U = 23.50, *p* = 0.05) at this point of assessment.

### 3.2. Cognitive Insight Trajectories in the Psychoeducation Group

#### 3.2.1. Self-Reflectivity

**Figure 3:**
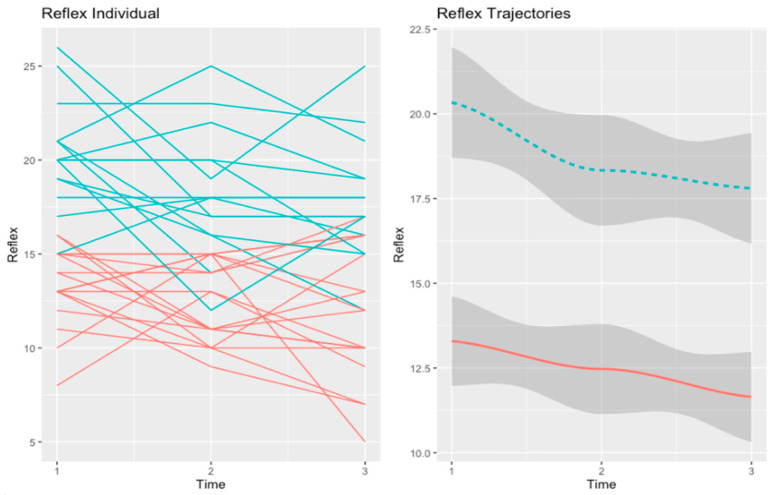
Trajectories of self-reflectivity in the psychoeducation group. The red line refers to the “Low-SR” trajectory (n = 18). The second trajectory (in blue), refers to the “Worsening-SR” (n = 16) trajectory.

In the psychoeducation group, we also found two trajectories for self-reflectivity. These are depicted in Figure 3.

Concerning self-reflectivity, the first trajectory (in red), henceforth “Low-SR” (n = 18), included patients that had a low baseline level of self-reflectivity. In this trajectory, self-reflectivity consistently declined over time. The second trajectory (in blue), henceforth “Worsening-SR” (n = 16), comprised patients exhibiting self-reflectivity that was high at baseline but declined steeply over time.

At baseline, the two trajectories of self-reflectivity differed in diagnosis (χ2 = 9.86, *p* = 0.04). Participants in the “Low-SR” trajectory had a bigger proportion of patients diagnosed with schizophrenia as opposed to other diagnoses in the spectrum of psychosis.

At post-test, participants in the “Low-SR” trajectory reported more personalizing bias (U = 47.00, *p* = 0.05). However, this difference was not maintained at follow-up. At the follow-up point of assessment, the two trajectories of self-reflectivity in the psychoeducation group did not differ in any variable.

#### 3.2.2. Self-Certainty

**Figure 4:**
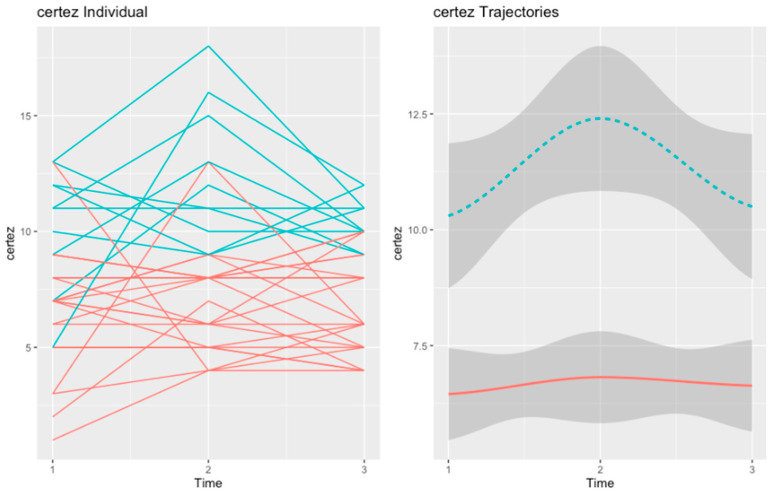
Trajectories of self-certainty in the psychoeducation group. In red is depicted the “Low and Stable SC” (n = 23) trajectory. The second trajectory, in blue, refers to the “Worsening SC” (n = 11) trajectory.

Two trajectories of self-certainty were apparent in the psychoeducation group. The first trajectory (in red), named “Low and Stable SC” (n = 23), included patients with low baseline self-certainty, which remained stable throughout the three assessments. The second trajectory (in blue), named “Worsening SC” (n = 11), included patients with high baseline self-certainty, which increased significantly after intervention and then decreased to baseline levels at follow-up. The graphical representation of these trajectories can be found in Figure 4.

At baseline, the two trajectories of self-certainty differed in academic background (χ2 = 11.98, *p* = 0.035), positive symptoms (U = 70.50, *p* = 0.040), clinical insight (U = 31.50, *p* < 0.001), the 60–40 condition of the beads task (U= 57.50, *p* = 0.011), and facial emotion recognition (U = 64.00, *p* = 0.019).

At post-test, the Worsening SC trajectory presented more positive (U = 27.50, *p* = 0.014) and negative (U = 34.50, *p* = 0.029) symptoms, worse global functioning (U = 37.50, *p* = 0.042), worse clinical insight (U = 33.00, *p* = 0.003), and less draws to decision in the three conditions of the beads task (U = 34.00, *p* = 0.023; U = 32.50, *p* = 0.021; U = 38.50, *p* = 0.043).

However, at follow up, the two trajectories only differed in positive (U = 25.00, *p* = 0.009) and negative symptoms (U = 30.00, *p* = 0.020), clinical insight (U = 30.50, *p* = 0.005), and theory of mind (U = 38.50, *p* = 0.05).

## 4. Discussion

As predicted by previous literature [18], we found that patients that received MCT improved cognitive insight by maintaining good baseline levels of self-reflectivity or improving low baseline levels and reducing self-certainty. This was an expected result since the original study [11] found solid evidence of the improvement in cognitive insight in patients who received MCT compared to those who received psychoeducation. By using LCMMs, we could obtain a deeper view of how patients respond to each intervention.

In absence of a specific intervention on cognitive insight, it seems that self-reflectivity tends to decrease steadily independent of its baseline level, which was evidenced by the two trajectories in the psychoeducation group. This finding is consistent with a previous study comparing psychoeducation and metacognitive training that found that psychoeducation is not an effective treatment to improve self-reflectivity [35].

Our results showed that metacognitive training is useful in maintaining high baseline self-reflectivity or improving low baseline self-reflectivity. However, trajectory analysis suggests that self-reflectivity tends to decrease after the intervention. In this sense, LCMMs allowed us to detect that MCT may be beneficial in improving cognitive insight for all patients, regardless of their baseline levels. Furthermore, we found that both self-reflectivity and self-certainty experienced a steep decline at follow-up, which suggests the need for maintained booster sessions of MCT to stabilize the effects. These results are consistent with recent evidence showing that extended therapy could strengthen its positive effects [9,28].

A previous study has suggested sustained and sleeper effects of MCT in people with psychosis [27]. In this work, the authors found that improvements in delusions that were not significant at the post-test became significant at a three-year follow-up. Because our follow-up was at six months, we were not able to detect whether any trajectory experienced more sustained effects, or whether the trajectories may change over time as the sleeper effect becomes apparent.

There was a surprising finding. Psychoeducation seemed to be detrimental to patients with high self-certainty at baseline. At baseline, participants in this trajectory experienced more positive symptoms, less clinical insight, a bigger tendency to make hasty decisions (jumping to conclusions), and worse facial emotion recognition than their counterparts. These differences were not apparent in the self-certainty trajectories of the MCT group. Moreover, at post-test, patients in the Worsening SC group presented fewer draws to decision in the three conditions of the beads task, which suggests a more pronounced tendency to present the jumping-to-conclusions bias.

Furthermore, both self-certainty and the jumping-to-conclusions bias are strongly related to delusions [14,23], and clinical insight and facial emotion recognition are strong predictors of outcome and functioning in patients with psychosis [12,56]. Because this trajectory had worse scores in these domains, we speculate that it represents a group with a higher risk of relapse, although this should be tested in future studies.

Although the scope of this study precludes us from drawing conclusions on the possible mechanisms of worsening self-certainty in response to psychoeducation, our interpretation is that there may be an interaction between unawareness of the disease, positive symptoms, and poor facial emotion recognition. These patients also had poor clinical insight and thus may interpret information on the disease as threatening and react by jumping to the conclusion that their thoughts and experiences are certain. These results suggest that patients that have social cognitive and metacognitive difficulties may benefit from starting psychological treatment with a normalizing approach that reduces data-gathering biases and improves insight, such as MCT.

The findings of this study must be interpreted considering several limitations: First, the sample size in the two groups was small, rendering the study underpowered and limiting our ability to compare longitudinal outcomes. Similarly, the sample size of each trajectory precluded us from obtaining predictors for each trajectory. Finally, our follow-up data only extended to six months after the intervention, and we could not detect sleeper effects.

These limitations notwithstanding, our findings support the use of LCMMs to study cognitive biases in response to psychotherapy and highlight the heterogeneous nature of psychosis. Future studies including broader samples comparing more interventions will help detect the chances of responding to a specific intervention and detect precision treatment strategies to prescribe psychological treatment based on the individual characteristics of each person.

This study was not designed to identify predictors of outcomes after MCT. Rather, the present study aimed to offer proof-of-concept evidence of the added value of using LCMMs to improve our current knowledge of psychological interventions for psychosis. However, using LCMMs with larger samples may help detect what specific trajectories have better chances of responding to an intervention and what their predictors are and may identify potential moderators.

Furthermore, this approach is not only valid for people with psychosis, as most current psychological interventions for mental illness have sound theoretical foundations and strong evidence supporting their efficacy.

## 5. Conclusions

This work supports the added value of using LCMMs to understand how specific psychological interventions exert differential effects on cognitive biases while considering the variability of the patients’ responses. Specifically, we found that MCT improves cognitive insight in all participants with first-episode psychosis regardless of their baseline level. Conversely, psychoeducation does not affect cognitive insight and may trigger adverse effects in some patients. Finally, LCMMs could be a useful approach to detect predictors of response to different psychological treatments and to develop early targeted treatment for people with psychosis.

## Figures and Tables

**Table 1:** Baseline differences in sociodemographic, clinical, social cognitive, and metacognitive variables between the psychoeducation and the MCT groups.

	MCT (n = 28)		Psychoeducation (n = 34)			
	Mean/%	SD	Mean/%	SD	t/χ2	*p*
Age						
Sex (% males)	75.00		73.50		0.17	0.90
Number of hospital admissions						
Education (%)					4.665	0.46
Incomplete primary education	21.40		11.80			
Complete primary education	28.60		17.60			
Incomplete secondary education	10.70		20.60			
Complete secondary education	25.00		26.50			
Incomplete superior education	3.60		14.70			
Complete superior education	10.70		8.80			
Diagnosis (%)					4.929	0.43
Schizophrenia	46.40		55.90			
Non-specified psychotic disorder	21.40		11.80			
Schizoaffective disorder	3.60		11.80			
Delusional disorder	7.10		11.80			
Brief psychotic episode	10.70		11.80			
Schizophreniform disorder	7.10		8.80			
PANSS						
Positive symptoms	12.25	3.874	12.53	4.487	0.26	0.80
Negative symptoms	14.54	7.162	15.15	4.794	0.40	0.69
General symptoms	27.36	7.790	27.26	5.920	−0.05	0.96
Total	54.14	15.717	54.94	12.110	0.23	0.82
GAF	66.61	13.331	57.50	11.758	−2.86	0.01
SUMD global score	5.82	2.695	6.03	3.639	0.25	0.80
Beads task						
85–15	3.6296	3.85455	5.3529	4.27737	1.632	0.11
60–40	6.4444	3.99358	8.5294	5.16536	1.726	0.09
Affective	6.3704	4.50767	7.7941	4.11781	1.286	1.286
IPSAQ						
Externalizing bias	0.1481	3.44968	1.5588	3.85488	1.486	0.14
Personalizing bias	1.2569	0.72667	1.2183	0.50730	−0.24	0.81
Hinting task	4.7143	1.01314	4.6765	0.97610	−0.15	0.88
Rosenberg self-esteem	27.5714	6.42004	27.3824	5.03933	−0.13	0.90
Faces test	17.6786	1.82683	17.4706	1.39773	−0.51	0.61
Estimated premorbid IQ	99.4231	14.85442	96.7188	13.71421	−0.72	0.48

## Data Availability

The data presented in this study are available on request from the corresponding author.

## Supplementary Materials

The following supporting information can be downloaded at: https://www.mdpi.com/article/10.3390/healthcare10112155/s1, Table S1: Baseline, post-test, and follow-up characteristics of each trajectory in the MCT group; Table S2: Baseline, post-test, and follow-up characteristics of each trajectory in the psychoeducation group.
