# Supplementary material for: Heterogeneity in Response to MCT and Psychoeducation: A Feasibility Study Using Latent Class Mixed Models in First-Episode Psychosis
Source: Healthcare (Basel). 2022 Oct 28;10(11):2155. doi: 10.3390/healthcare10112155 (PMC9691095; doi:10.3390/healthcare10112155)
Supplement: Supplementary file 1 [file healthcare-10-02155-s001.zip › healthcare-1935267-supplementary.pdf]

Table S1. Baseline, post-test and follow up characteristics of each trajectory in the MCT group.

	Metacognitive Training Group													
	Self-reflectivity							Self-certainty						
	High SR (N=20)		Improving SR (N=8)					Initial decline (N=22)		Responsive SC (N=6)				
	**Mean/%**	**SD**	**Mean/%**	**SD**	**U/χ2**	**p**	**RBC**	**Mean/%**	**SD**	**Mean/%**	**SD**	**U/χ2**	**p**	**RBC**
**Baseline**														
Age	25.850	7.916	25.500	7.838	78.50	0.959	0.01875	24.455	6.262	30.500	11.185	46.00	0.273	0.30303
Number of hospital admissions	0.750	0.786	2.000	1.826	38.50	0.063	0.45000	0.952	1.024	1.500	1.871	54.50	0.613	0.13492
Education					2.829	0.726						9.934	0.077	
Incomplete primary education	15		37.5					22.7		16.7				
Complete primary education	35		12.5					31.8		16.7				
Incomplete secondary education	10		12.5					4.5		33.3				
Complete secondary education	25		25					31.8						
Incomplete superior education	5									16.7				
Complete superior education	10		12.5					9.1		16.7				
Diagnosis					4.035	0.544						2.077	0.838	
Schizophrenia	40		62.5					45.5		50				
Non-specified psychotic disorder	25		1					18.2		33.3				
Schizoaffective disorder	5							4.5		16.7				
Delusional disorder	5		1					9.1						
Brief psychotic episode	15							9.1						
Schizophreniform disorder	10							9.1						
PANSS														
Positive symptoms	12.400	4.512	11.875	1.553	77.50	0.919	0.03125	11.545	3.247	14.833	5.154	38.00	0.122	0.42424
Negative symptoms	12.050	5.898	20.750	6.453	20.00	0.002	0.75000	13.636	6.075	17.833	10.265	55.50	0.574	0.15909
General symptoms	24.600	6.613	34.250	6.274	22.00	0.003	0.72500	25.955	6.979	32.500	9.094	36.50	0.104	0.44697
Total	49.050	14.255	66.875	11.910	25.50	0.006	0.68125	51.136	13.361	65.167	19.964	37.00	0.110	0.43939
GAF	71.500	10.390	54.375	12.374	23.00	0.004	0.71250	68.409	12.565	60.000	15.166	42.50	0.193	0.35606
SUMD global score	5.600	2.542	6.375	3.159	68.50	0.561	0.14375	5.818	2.648	5.833	3.125	66.00	1.000	0.00000

Beads Task														
85-15	3.842	4.400	3.125	2.232	75.50	1.000	0.00658	3.857	4.269	2.833	1.835	60.00	0.881	0.04762
60-40	6.842	4.180	5.500	3.586	66.00	0.611	0.13158	6.571	4.142	6.000	3.742	61.00	0.930	0.03175
Affective	6.053	3.937	7.125	5.890	69.00	0.728	0.09211	6.571	4.760	5.667	3.777	60.00	0.883	0.04762
IPSAQ														
Externalizing bias	0.450	3.546	-0.714	3.251	57.50	0.504	0.17857	-0.238	3.548	1.500	2.950	44.00	0.277	0.30159
Personalizing bias	1.351	0.693	0.987	0.809	54.00	0.391	0.22857	1.224	0.801	1.373	0.398	58.00	0.793	0.07937
Hinting task	4.850	0.745	4.375	1.506	68.50	0.532	0.14375	4.682	0.995	4.833	1.169	59.50	0.708	0.09848
Rosenberg self-esteem	29.700	4.932	22.250	6.902	29.50	0.011	0.63125	26.636	6.807	31.000	3.162	38.50	0.130	0.41667
Faces Test	17.700	2.003	17.625	1.408	73.50	0.757	0.08125	17.955	1.676	16.667	2.160	42.50	0.191	0.35606
Estimated premorbid IQ	101.000	15.778	94.167	10.685	44.50	0.354	0.25833	100.250	15.515	96.667	13.292	50.50	0.578	0.15833
**Post-test**														
PANSS														
Positive symptoms	10.842	3.594	12.000	1.414	42.00	0.352	0.26316	10.158	2.544	14.167	3.430	18.50	0.015	0.67544
Negative symptoms	11.737	5.425	21.500	7.556	12.00	0.004	0.78947	13.211	6.503	16.833	9.261	46.00	0.501	0.19298
General symptoms	24.263	6.244	32.667	6.346	21.50	0.026	0.62281	25.053	6.442	30.167	8.472	36.50	0.202	0.35965
Total	46.842	13.297	66.167	11.161	13.50	0.006	0.76316	48.421	13.285	61.167	17.837	33.00	0.134	0.42105
GAF	69.895	8.627	57.500	12.942	20.50	0.021	0.64035	67.263	11.780	65.833	8.612	49.50	0.653	0.13158
SUMD global score	4.667	2.301	6.667	1.966	24.00	0.040	0.55556	5.167	2.307	5.167	2.714	66.00	1.000	0.00000
Beads Task														
85-15	5.474	5.491	4.200	1.095	42.50	0.742	0.10526	5.611	5.564	4.000	1.789	60.00	0.881	0.04762
60-40	8.684	5.154	9.000	2.646	42.50	0.747	0.10526	9.278	4.824	7.167	4.262	61.00	0.930	0.03175
Affective	8.158	4.610	7.200	3.114	47.00	1.000	0.01053	8.111	4.310	7.500	4.637	60.00	0.883	0.04762
IPSAQ														
Externalizing bias	0.158	2.410	1.400	1.817	31.50	0.265	0.33684	0.778	2.315	-0.667	2.160	44.00	0.277	0.30159
Personalizing bias	1.254	0.460	0.958	0.529	34.00	0.354	0.28421	1.178	0.496	1.236	0.466	58.00	0.793	0.07937
Hinting task	5.158	1.015	4.500	2.258	50.00	0.657	0.12281	4.895	1.449	5.333	1.211	59.50	0.708	0.09848
Rosenberg self-esteem	29.368	4.512	23.167	8.565	27.00	0.059	0.52632	26.895	6.616	31.000	2.966	38.50	0.130	0.41667
Faces Test	17.211	2.070	17.667	1.506	54.50	0.897	0.04386	17.421	1.774	17.000	2.530	42.50	0.191	0.35606

**6 month FU**														
PANSS														
Positive symptoms	10.333	3.694	11.333	2.422	37.00	0.263	0.31481	9.556	2.455	13.667	4.179	20.50	0.025	0.62037
Negative symptoms	11.278	5.399	19.000	9.143	22.00	0.034	0.59259	12.389	6.705	15.667	8.595	43.00	0.479	0.20370
General symptoms	21.722	5.027	32.400	7.987	10.00	0.010	0.77778	22.294	6.008	29.000	8.414	23.50	0.058	0.53922
Total	43.333	12.705	65.000	17.507	12.50	0.017	0.72222	44.412	14.444	58.333	17.941	24.50	0.068	0.51961
GAF	73.444	9.482	65.167	16.678	33.50	0.178	0.37963	71.278	12.352	71.667	11.255	52.50	0.946	0.02778
SUMD global score	4.444	1.756	7.500	3.017	20.00	0.020	0.62963	5.167	2.595	5.333	2.251	49.00	0.756	0.09259
Beads Task														
85-15	4.778	1.768	3.167	2.041	30.50	0.119	0.43519	4.222	2.130	4.833	1.169	40.50	0.378	0.25000
60-40	8.222	5.242	6.667	4.082	43.50	0.503	0.19444	7.167	4.541	9.833	5.981	39.00	0.331	0.27778
Affective	7.667	3.646	6.833	3.488	49.00	0.761	0.09259	7.444	3.944	7.500	2.258	50.50	0.839	0.06481
IPSAQ														
Externalizing bias	1.944	3.404	0.800	3.114	37.50	0.598	0.16667	1.941	3.526	1.000	2.757	42.50	0.572	0.16667
Personalizing bias	1.275	0.317	1.097	0.403	7.00	0.357	0.41667	1.369	0.215	0.978	0.379	6.00	0.155	0.57143
Hinting task	5.111	1.023	4.667	1.366	42.00	0.413	0.22222	5.000	1.085	5.000	1.265	51.50	0.887	0.04630
Rosenberg self-esteem	31.333	3.926	25.833	8.565	31.50	0.141	0.41667	29.722	6.304	30.667	4.131	53.00	0.973	0.01852
Faces Test	17.611	1.787	18.333	1.211	41.50	0.409	0.23148	17.889	1.023	17.500	3.017	50.00	0.810	0.07407

Table S2. Baseline, post-test, and follow-up characteristics of each trajectory in the psychoeducation group.

	Psychoeducation													
	Self-reflectivity							Self-certainty						
	Low SR (N=17)		Worsening SR (N=16)					Low and stable SC (N=23)		Worsening SC (N=11)				
	**Mean/%**	**SD**	**Mean/%**	**SD**	**U/χ2**	**p**	**RBC**	**Mean/%**	**SD**	**Mean/%**	**SD**	**U/χ2**	**p**	**RBC**
**Baseline**														
Age	29.235	6.300	28.188	7.884	123.00	0.652	0.09559	29.304	6.131	27.818	8.623	110.50	0.568	0.12648
Number of hospital admissions	1.882	1.409	1.313	1.138	107.50	0.299	0.20956	1.478	1.310	1.818	1.250	106.00	0.447	0.16206
Education					2.102	0.835						11.98	0.035	
Incomplete primary education	11.8		12.5					8.7		18.2				
Complete primary education	11.8		18.8					13		27.3				
Incomplete secondary education	29.4		12.5					8.7		45.5				
Complete secondary education	23.5		31.3					34.8		9.1				
Incomplete superior education	11.8		18.8					21.7						
Complete superior education	11.8		6.3					13						
Diagnosis					9.86	0.04						3.9	0.42	
Schizophrenia	76.5		31.3					52.2		63.3				
Non-specified psychotic disorder			25					13		9.1				
Schizoaffective disorder	5.9		18.8					13		9.1				
Delusional disorder														
Brief psychotic episode	5.9		18.8					17.4						
Schizophreniform disorder	11.8		6.3					4.3		18.2				
PANSS														
Positive symptoms	13.529	4.571	11.813	4.277	106.00	0.285	0.22059	11.478	4.491	14.727	3.771	70.50	0.040	0.44269
Negative symptoms	16.235	4.790	13.750	4.655	97.00	0.164	0.28676	14.478	4.571	16.545	5.165	100.50	0.346	0.20553
General symptoms	28.000	5.500	26.563	6.603	113.00	0.416	0.16912	26.304	6.018	29.273	5.424	93.00	0.223	0.26482
Total	57.765	11.306	52.125	12.971	97.00	0.165	0.28676	52.261	12.531	60.545	9.353	78.50	0.080	0.37945
GAF	55.588	11.974	59.375	11.955	125.00	0.702	0.08088	59.565	12.605	53.182	8.739	78.00	0.074	0.38340
SUMD global score	6.706	3.567	5.500	3.759	107.50	0.283	0.20956	4.391	2.589	9.455	3.142	31.50	< .001	0.75099

Beads Task														
85-15	6.294	5.312	4.500	2.828	117.50	0.511	0.13603	5.696	4.072	4.636	4.802	88.00	0.155	0.30435
60-40	9.647	5.338	7.375	5.045	106.00	0.286	0.22059	9.913	5.080	5.636	4.202	57.50	0.011	0.54545
Affective	8.706	4.858	6.750	3.152	116.50	0.490	0.14338	8.391	3.615	6.545	4.967	82.50	0.106	0.34783
IPSAQ														
Externalizing bias	1.176	4.290	2.000	3.559	111.00	0.374	0.18382	2.087	4.078	0.455	3.236	111.50	0.591	0.11858
Personalizing bias	1.365	0.533	1.084	0.460	100.00	0.201	0.26471	1.219	0.556	1.218	0.411	125.50	0.985	0.00791
Hinting task	4.647	1.057	4.688	0.946	132.50	0.906	0.02574	4.783	0.951	4.455	1.036	106.50	0.427	0.15810
Rosenberg self-esteem	28.000	4.690	17.647	1.455	125.50	0.718	0.07721	27.391	4.283	27.364	6.592	125.50	0.985	0.00791
Faces Test	17.647	1.455	17.188	1.328	118.50	0.529	0.12868	17.870	1.254	16.636	1.362	64.00	0.019	0.49407
Estimated premorbid IQ	96.563	10.912	97.667	16.676	112.50	0.779	0.06250	98.409	14.090	93.000	12.737	86.00	0.333	0.21818
**Post-test**														
PANSS														
Positive symptoms	11.462	3.230	9.667	3.172	49.00	0.118	0.37179	9.556	2.640	13.000	3.207	27.50	0.014	0.61806
Negative symptoms	14.769	4.304	13.385	4.664	67.00	0.381	0.20710	13.000	4.346	16.750	3.327	34.50	0.029	0.54605
General symptoms	24.385	4.735	24.385	3.686	81.50	0.898	0.03550	23.737	3.813	26.375	4.534	50.50	0.182	0.33553
Total	50.615	10.540	46.583	7.821	62.00	0.398	0.20513	45.667	8.196	56.125	7.434	24.00	0.008	0.66667
GAF	61.846	10.900	62.846	11.149	80.00	0.836	0.05325	64.789	10.753	56.250	7.906	37.50	0.042	0.50658
SUMD global score	5.308	3.521	4.077	2.660	65.50	0.224	0.22485	3.421	1.427	7.500	4.071	33.00	0.003	0.56579
Beads Task														
85-15	4.000	2.677	5.846	5.336	73.00	0.563	0.13609	5.789	4.590	2.625	1.598	34.00	0.023	0.55263
60-40	8.462	4.502	8.385	2.873	81.50	0.897	0.03550	9.737	3.034	5.625	3.543	32.50	0.021	0.57237
Affective	7.923	4.734	7.385	2.468	84.00	1.000	0.00592	8.684	3.146	5.375	3.889	38.50	0.043	0.49342
IPSAQ														
Externalizing bias	0.231	3.609	1.692	5.154	73.00	0.571	0.13609	0.895	4.748	1.750	3.845	63.50	0.522	0.16447
Personalizing bias	1.375	0.459	1.092	0.468	47.00	0.057	0.44379	1.232	0.567	1.137	0.255	65.50	0.595	0.13816
Hinting task	5.077	1.256	4.538	1.613	70.00	0.447	0.17160	5.053	1.471	4.375	1.302	45.00	0.085	0.40789
Rosenberg self-esteem	29.231	3.516	30.462	5.125	75.50	0.661	0.10651	29.737	4.629	29.250	4.334	74.50	0.957	0.01974
Faces Test	17.462	1.450	18.154	1.214	61.50	0.233	0.27219	18.000	1.291	17.500	1.512	62.00	0.457	0.18421

**6 month FU**														
PANSS														
Positive symptoms	11.385	3.686	9.750	3.415	51.00	0.144	0.34615	9.556	3.072	13.375	3.335	25.00	0.009	0.65278
Negative symptoms	13.923	4.804	12.167	4.152	57.50	0.274	0.26282	11.667	3.614	16.125	4.704	30.00	0.020	0.58333
General symptoms	24.154	4.079	22.917	3.204	58.00	0.286	0.25641	22.647	3.622	25.500	3.117	38.50	0.089	0.43382
Total	49.462	10.170	44.833	7.918	55.50	0.231	0.28846	43.588	8.389	55.000	5.732	19.50	0.005	0.71324
GAF	63.846	12.825	65.000	12.060	73.00	0.805	0.06410	66.667	12.166	58.750	10.264	44.00	0.124	0.38889
SUMD global score	5.385	3.618	4.250	2.989	67.50	0.511	0.13462	3.500	1.689	7.625	4.207	30.50	0.005	0.57639
Beads Task														
85-15	4.769	4.003	4.750	3.864	68.50	0.619	0.12179	4.778	3.318	4.625	4.926	50.50	0.236	0.29861
60-40	7.692	4.461	8.083	3.175	63.00	0.426	0.19231	8.500	2.854	6.625	5.263	47.50	0.179	0.34028
Affective	7.692	4.956	7.833	2.980	68.50	0.622	0.12179	8.278	2.718	6.750	6.042	44.50	0.131	0.38194
IPSAQ														
Externalizing bias	1.231	3.539	2.250	3.957	68.00	0.604	0.12821	2.722	3.528	0.000	3.665	43.00	0.112	0.40278
Personalizing bias	1.663	1.660	0.956	0.767	10.00	0.429	0.33333	1.007	0.545	2.845	3.048	8.00	0.747	0.20000
Hinting task	5.000	0.913	4.583	0.996	59.00	0.287	0.24359	5.056	0.802	4.250	1.035	38.50	0.051	0.46528
Rosenberg self-esteem	28.615	5.853	29.167	5.424	71.50	0.743	0.08333	29.667	5.541	26.125	5.410	49.00	0.210	0.31944
Faces Test	17.846	1.725	17.750	1.288	74.50	0.868	0.04487	18.000	1.328	17.500	1.852	59.00	0.479	0.18056
